# Accelerated Loss of TCR Repertoire Diversity in Common Variable Immunodeficiency

**DOI:** 10.4049/jimmunol.1600526

**Published:** 2016-08-01

**Authors:** Gabriel K. Wong, David Millar, Sarah Penny, James M. Heather, Punam Mistry, Nico Buettner, Jane Bryon, Aarnoud P. Huissoon, Mark Cobbold

**Affiliations:** * Medical Research Council Centre for Immune Regulation, University of Birmingham, Edgbaston B15 2TT, United Kingdom;; † West Midlands Primary Immunodeficiency Centre, Birmingham Heartlands Hospital, Birmingham B9 5SS, United Kingdom;; ‡ Cancer Center, Massachusetts General Hospital, Boston MA 02114;; § Department of Medicine, Harvard Medical School, Charlestown, MA 02129;; ¶ Division of Infection and Immunity, University College London, London WC1E 6BT, United Kingdom; and; ‖ Regional Genetics Laboratory, Birmingham Women’s Hospital, Birmingham B15 2TG, United Kingdom

## Abstract

Although common variable immunodeficiency (CVID) has long been considered as a group of primary Ab deficiencies, growing experimental data now suggest a global disruption of the entire adaptive immune response in a segment of patients. Oligoclonality of the TCR repertoire was previously demonstrated; however, the manner in which it relates to other B cell and T cell findings reported in CVID remains unclear. Using a combination approach of high-throughput TCRβ sequencing and multiparametric flow cytometry, we compared the TCR repertoire diversity between various subgroups of CVID patients according to their B cell immunophenotypes. Our data suggest that the reduction in repertoire diversity is predominantly restricted to those patients with severely reduced class-switched memory B cells and an elevated level of CD21^lo^ B cells (Freiburg 1a), and may be driven by a reduced number of naive T cells unmasking underlying memory clonality. Moreover, our data indicate that this loss in repertoire diversity progresses with advancing age far exceeding the expected physiological rate. Radiological evidence supports the loss in thymic volume, correlating with the decrease in repertoire diversity. Evidence now suggests that primary thymic failure along with other well-described B cell abnormalities play an important role in the pathophysiology in Freiburg group 1a patients. Clinically, our findings emphasize the integration of combined B and T cell testing to identify those patients at the greatest risk for infection. Future work should focus on investigating the link between thymic failure and the severe reduction in class-switched memory B cells, while gathering longitudinal laboratory data to examine the progressive nature of the disease.

## Introduction

The Ag receptor repertoire is a distinctive feature of the adaptive immune system that enables the recognition of a vast potential of Ags. The process of Ag receptor gene rearrangement (VDJ) provides each T lymphocyte its own unique Ag binding characteristic and molecular signature, each contributing to the tremendous diversity within our immune repertoires (1). The diversity of a T cell immune repertoire is maintained by continuous outputs from the thymus, although it evidently diminishes as part of the normal aging process (2).

Common variable immunodeficiency (CVID) has long been considered as a primary Ab deficiency, although growing experimental data now suggest a global disruption of the entire adaptive immune response. Assessments of the IgH and TCR repertoires in the past have demonstrated gross abnormalities in both B cell and T cell compartments (3–5).

It has been proposed that the observed T cell oligoclonality in CVID is predominantly driven by the abnormal expansion of CD8^+^ T cells, with an unexplained bias toward particular V-genes dominating the TCR repertoire, although a similar, albeit distinct, pattern has also been observed in CD4 T cells (4–6). CD8 oligoclonality appears to be unique to CVID, because age-matched X-linked agammaglobulinemia patients tend to have normal diversity in their CD8 repertoire (4). To further support the overexpansion of CD8^+^ T cells, replicative senescence has been reported in the T cells of CVID patients (7). In addition, immunophenotypic data have consistently demonstrated the increased expression of CD95, CD38, and HLA-DR along with elevated levels of CD57 and PD-1 on CD8^+^ T cells, indicating their active inflammatory role with evidence of exhaustion (8–11). Given closer HLA sharing among CVID patients (12, 13), the combination of Vβ gene restriction and an activated CD8 phenotype suggests the presence of common antigenic drivers behind their expansion.

Large cohort studies have revealed, however, that only a small proportion of patients have elevated peripheral absolute CD8^+^ T cell counts (8, 14). Importantly, a reversed CD4:CD8 ratio was often a result of CD4 lymphopenia without dramatic alteration in the absolute CD8^+^ T cell count (8, 14). Hence the overall influences on the repertoire diversity of CD8^+^ T cells remain unexplained, and may be restricted solely to a subgroup of patients.

Recent evidence using more powerful molecular approaches suggests that the restriction of the TCR repertoire in CVID may be related to an intrinsic defect, such as reduced junctional diversity and V-region deletion, as opposed to a response to an extrinsic chronic antigenic driver (3), thus revising our understanding of CVID as a combined T cell and B cell immunodeficiency. Nevertheless, the reported intrinsic repertoire defect was relatively subtle, with many patients carrying a normal TCR repertoire. Given the known heterogeneity of CVID, we hypothesize that immune repertoire defects are restricted to a subgroup of patients, and that analysis according to an internationally agreed classification would facilitate in relating the repertoire defect to previously described experimental findings with greater accuracy.

This study describes and compares the TCR repertoire diversity between subgroups of CVID patients according to their B cell immunophenotypes (15). Using a combination approach of high-throughput TCR sequencing and multiparametric flow cytometry, our data suggest that a loss of TCR repertoire diversity is predominantly found in Freiburg 1a patients in a progressive manner. Consistent with previous reports, a reduction of naive T cells appeared to be a driver for oligoclonality and increased risk for infection (5, 10); in contrast, the role of effector memory CD8^+^ T cells in altering the repertoire diversity is less apparent.

## Materials and Methods

### Participants and ethics

This study was conducted according to protocols approved by the South Birmingham Research Ethics Committee, National Research Ethics Service, U.K. (REC reference: 11/WM/0041), and was sponsored by the University of Birmingham (Reference: RG_10-260). Written consent was obtained from all participants in accordance with the Declaration of Helsinki. Forty-two patients with CVID were recruited from the West Midlands Immunodeficiency Centre, U.K., all of whom were diagnosed according to the European Society of Immunodeficiencies and Pan American Group for Immunodeficiency criteria. The CVID patients were subclassified according to their peripheral blood B cell subsets (<1% peripheral B cells, Freiburg 1a = <0.4% switched memory B cells of PBLs and >20% CD21^lo^ B cells of total B cells, Freiburg 1b = <0.4% switched memory B cells and <20% CD21^lo^ B cells, and Freiburg 2 = >0.4% switched memory B cells) (15). The patients’ characteristics are given in Supplemental Table I. Clinical, laboratory, and radiological data were collated with the findings of this study. Finally, age-matched healthy donors were recruited from within the University of Birmingham as control subjects.

### Next-generation sequencing of TCRβ repertoire

The genomic DNA of the healthy donors and CVID patients were obtained by extraction from 2.5 × 10^6^ PBMCs using the ISOLATE II Genomic DNA Kit (Bioline) or DNeasy Blood & Tissue Kit (Qiagen) according to the standard manufacturer’s protocols. The TCRβ repertoires were assessed via the ClonoSIGHT platform (Sequenta) as previously described (16). All raw antisense nucleotide TCRβ sequences obtained were reverified and converted to amino acid TCRβ sequences through the IMGT online-based bioinformatics tool (http://www.imgt.org), HighV-Quest, under the following settings: species = human; locus = TCRβ; single individual, nucleotide per line in alignments = 60; aligned reference sequences = 5. Details of sequencing output are provided in Supplemental Table II.

### T cell repertoire diversity and V-gene usage

Relative repertoire diversity was estimated by Shannon entropy (S.E.; natural log: ln) [H = −Σ p(x) ln p(x); p(x) = frequency of individual amino acid TCRβ sequences] and inverse Simpson index [1/λ = 1/Σ p(x)^2^] as previously described (17–19), with only the in-frame productive TCRβ sequences being considered in the calculation. Higher scores were generated for polyclonal samples, whereas a lower score indicated clonality. V-gene usages of all subgroups were compared by Jenson–Shannon divergence (JSD) (20).

### Multiparametric flow cytometry

A whole blood staining protocol was used for the peripheral blood T cell immunophenotyping. Four hundred fifty milliliters of EDTA blood was labeled with anti–CD3-PE.Cy5, anti–CD4-BV711, anti–CD8a-allophycocyanin, anti–CD45RA-BV510, anti–CD27-BV605, anti–CD28-PE/Cy7, anti–CCR7-PE/Cy5.5, and anti-CD25-allophycocyanin/Cy7 Abs (Biolegend) to determine various T cell subpopulations. Abs were used in 1:100 dilutions and incubated at room temperature for 20 min in the dark. All cells were washed with isotonic PBS with 2% FCS and incubated with 2 ml of 0.16 M of NH_4_CL at room temperature in the dark for 10 min for red cell lysis. A final wash with PBS and 2% FCS was carried out, and 0.5 ml of 1% paraformaldehyde was added. CountBright Absolute Counting Beads (Life Technologies) were added according to manufacturer’s protocol for accurate cell counting. Compensation was aided by the Anti-Mouse Ig, κ/Negative Control Compensation Particles Set (BD Biosciences) and automatically calculated by BD FACSDiva software version 6.0. Acquisition was performed via BD LSR Fortessa flow cytometer (BD Biosciences) and analyzed via FlowJo version 7.6.5 software.

### Statistical analysis

Statistical analyses were performed through GraphPad Prism version 5.0. Comparisons between nonparametric data sets were done via the two-tailed Mann–Whitney *U* test, whereas analysis of covariance (ANCOVA) and Kruskal–Wallis test were done via IBM SPSS statistic 21. The *p* values considered to be statistically significant were: *p < 0.05, **p < 0.01, and ***p < 0.001.

## Results

### Accelerated reduction in T cell repertoire diversity in Freiburg 1a patients

**FIGURE 1.**
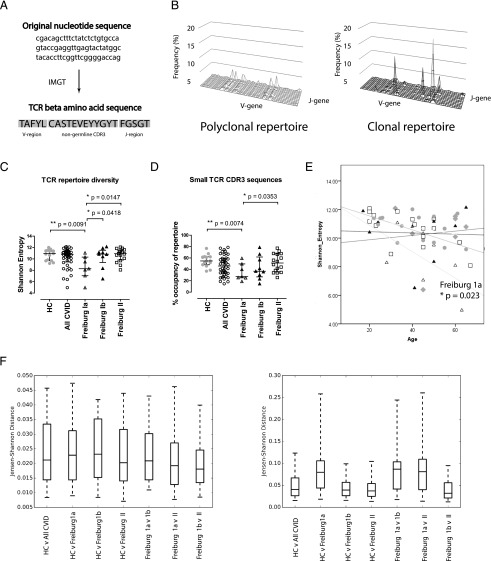
Next-generation sequencing of TCRβ-chain and assessment of repertoire diversity. TCR deep sequencing was carried out on the PBMCs of healthy donors and CVID patients according to the ClonoSIGHT platform. (**A**) Raw nucleotide sequences were converted into amino acid TCRβ sequences using IMGT’s HighV-Quest (left). (**B**) Graphic examples of a polyclonal (left) and a clonal (right) repertoire are shown according to the relative usage of V- and J-gene segments. (**C** and **D**) Repertoire diversity by S.E. and amino acid TCRβ sequences of <0.001% were compared among healthy donors (*n* = 15, gray circle), all CVID patients (*n* = 42, open circle), Freiburg group 1a patients (*n* = 7, open triangle), group 1b patients (*n* = 9, black triangle), and group 2 patients (*n* = 16, open square). (**E**) Repertoire diversity versus age is shown. JSDs of unweighted (**F**) and weighted (**G**) intragroup analyses (HC versus all CVID, HC versus Freiburg 1a, HC versus Freiburg 1b, HC versus Freiburg 2, Freiburg 1a versus Freiburg 1b, Freiburg 1a versus Freiburg 2, and Freiburg 1b versus Freiburg 2) are shown. The medians and interquartile ranges are depicted. Statistical differences highlighted by *, **, or *** (two-tailed Mann–Whitney *U* test and ANCOVA).

TCRβ next-generation sequencing was carried out to determine the level of TCR diversity in the CVID patients by using the genomic DNA extracted from the PBMCs. All nucleotide sequences were verified by the HighV-Quest tool to derive their V-J family usages and nongermline CDR3 amino acid sequences (Fig. 1A). Only productive sequences were considered for further analysis. Graphical examples of a polyclonal and a clonal TCR repertoire are shown in Fig. 1B, with distinct and abnormal peaks presenting in the clonal repertoire, but not in the polyclonal repertoire. TCR repertoire diversity was compared using S.E. (Fig. 1C), inverse Simpson’s diversity index, and cumulative percentage of the top 10 CDR3 sequences (Supplemental Fig. 1A, 1B). All three metrics demonstrated simple findings. According to Warnatz and colleagues (15), the CVID patients were classified into Freiburg group 1a (<0.4% class-switched memory B cells in peripheral blood, >20% CD21^lo^ B cells), Freiburg group 1b (<0.4% class-switched memory B cells in peripheral blood, <20% CD21^lo^ B cells), and Freiburg group 2 (>0.4% class-switched memory B cells). Age-matched healthy donors were recruited as control subjects (median age: healthy control [HC] = 43, all CVID = 45, Freiburg 1a = 45, Freiburg 1b = 41, Freiburg 2 = 40.5). The CVID patients as a whole demonstrated normal TCR repertoire diversity when compared with the healthy donors, whereas a significant decrease was detected in the Freiburg 1a patients (*p* = 0.009). By contrast, normal repertoire diversities were detected in the Freiburg 1b and Freiburg 2 patients.

To detect the potential source of oligoclonality in the Freiburg 1a patients, we divided the amino acid TCRβ sequences into low frequency (small: <0.001%), medium (0.001–0.01%), large (0.01–1%), or hyperexpanded (>1%), and compared their percentage occupancies in the repertoire between various subgroups of patients (2). Our data showed a significant reduction in low-frequency (<0.001%) amino acid TCRβ sequences uniquely in Freiburg 1a patients, but no other groups (HC versus Freiburg 1a, *p* = 0.007; Fig. 1D), whereas the overall occupancies of medium-to-hyperexpanded TCRβ sequences were unaffected (Supplemental Fig. 1C). The normality of the medium-to-hyperexpanded compartments strongly suggests a loss of low-frequency (<0.001%) TCRβ sequences as the source of oligoclonality. Furthermore, accelerated loss in repertoire diversity was noted with advancing age in the Freiburg 1a patients when compared with the other groups (ANCOVA, *p* = 0.023; Fig. 1E).

JSD was used to compare V-gene usage between subgroups of patients. Unweighted TCR sequences, where the frequencies or reads of individual sequences were not considered, showed no differences in V-gene usage between all groups (Fig. 1F). By contrast, JSD of weighted TCR sequences, where the frequencies of individual sequences were accounted for, demonstrated greater distance of Freiburg 1a patients from the other groups, indicating abnormal V-gene selection after clonal expansion (Fig. 1G). Finally, the numbers of P and N nucleotide insertion and deletion by both weighted and unweighted analyses were similar between all groups, with only a marginal increase in unweighted V, D, and J segment deletion observed in Freiburg 1a patients (Supplemental Fig. 1D, 1E).

Overall, our data indicate that the progressive loss of low-frequency (<0.001%) TCRβ sequences may reduce the repertoire diversity in Freiburg 1a patients, with evidence of disparate V-gene selection for clonal expansion.

### Reduction in naive T cells correlates with decreased repertoire diversity

**FIGURE 2.**
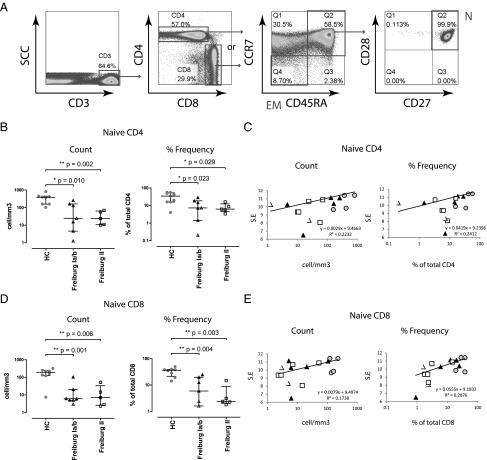
Correlations between repertoire diversity and peripheral naive T cells. Peripheral blood T cell subpopulations were enumerated using counting beads via multiparametric flow cytometry. (**A**) An example of the gating strategy is presented. (**B**) Naive (N:CCR7^+^CD45RA^+^CD28^+^CD27^+^) CD4 T cell counts and frequencies of healthy donors (*n* = 8, gray), Freiburg group 1a patients (*n* = 2, open triangle), group 1b patients (*n* = 5, black triangle), and group 2 patients (*n* = 5, open square) are shown. (**C**) Naive CD4 T cell counts and frequencies are plotted against TCRβ repertoire diversity (S.E.). The black lines represent the best fit line with *r*^2^ depicted adjacently. (**D**) Naive CD8 T cell counts and frequencies of healthy donors, CVIDs, Freiburg group 1a patients, group 1b patients, and group 2 patients are shown. (**E**) Naive CD8 T cell counts and frequencies are plotted against TCRβ repertoire diversity. Medians and interquartile ranges are depicted. Statistical differences are highlighted by *, **, or *** (two-tailed Mann–Whitney *U* test).

Given that low-frequency (<0.001%) TCRβ sequences are likely to represent unexpanded naive T cells, the correlation between naive T cell count and TCRβ repertoire diversity was further examined. CD4^+^ and CD8^+^ naive T cells (CCR7^+^CD45RA^+^CD28^+^CD27^+^) were determined by multiparametric flow cytometry and accurately calculated via counting beads (Fig. 2A). A good correlation was observed between the percentage of naive T cells and the percentage of low-frequency (small) TCR sequences, indicating that low-frequency TCR sequences were representative of the unexpanded naive pool (Supplemental Fig. 2A).

Reduced naive CD4^+^ T cell frequency and absolute count were seen in all subgroups of CVID patients (median cell count: HC = 384 mm^3^, Freiburg 1a/b = 77 mm^3^ [*p* = 0.01], Freiburg 2 = 24 mm^3^ [*p* = 0.002]; median frequency: HC = 38.0%, Freiburg 1a/b = 10.9% [*p* = 0.023], Freiburg 2 = 6.2% [*p* = 0.029]), with a positive correlation found between naive CD4^+^ T cells and S.E. repertoire diversity (Fig. 2B, 2C). A similar magnitude of naive CD8^+^ T cell decrease was also observed in all patients, where a positive correlation was demonstrated with repertoire diversity (median cell count: HC = 193 mm^3^, Freiburg 1a/b = 6 mm^3^ [*p* = 0.001], Freiburg 2 = 7 mm^3^ [*p* = 0.006]; median frequency: HC = 36.2%, Freiburg 1a/b = 9.2% [*p* = 0.004], Freiburg 2 = 2.4% [*p* = 0.003]) (Fig. 2D, 2E). A good linear correlation between the frequencies of naive CD4 and naive CD8 T cells could be seen, which supports a global deficiency in the naive T cell pool (Supplemental Fig. 2B).

In contrast, other T cell subpopulation counts such as effector memory (CCR7^−^CD45RA^−^), central memory (CCR7^+^CD45RA^−^CD27^+^CD28^+^), and terminally differentiated memory T cells (TEMRA: CCR7^−^CD45RA^+^) were largely normal, suggesting that the T cell memory compartments in CVID are relatively less affected (Supplemental Fig. 2C).

### Reduction in thymic volume in Freiburg 1a/b patients

**FIGURE 3.**
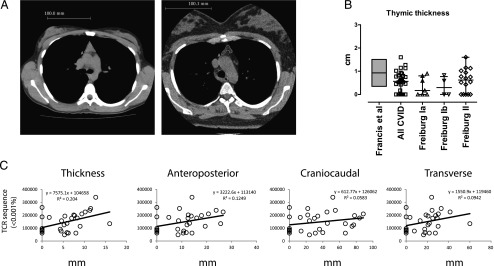
Thymic dimensions in CVID. Thymic size was assessed using a high-resolution CT scan and comparing this with an age-matched published reference range and repertoire diversity. (**A**) Examples of a full thymus (left: 20-year-old CVID patient) and a small hypodense thymus (right: 29-year-old CVID patient) are shown. (**B**) The thymic thickness (in centimeters) of CVID (open square, *n* = 30), Freiburg group 1a (open triangle, *n* = 7), Freiburg group 1b (open inverted triangle, *n* = 4), and Freiburg group 2 patients (open diamond, *n* = 16), and the expected range of individuals between 40 and 50 y of age (gray; mean and SD) obtained from Francis et al. (21) are presented. (**C**) Thymic dimensions (thickness, anteroposterior, craniocaudal, and transverse lengths in centimeters) are plotted against repertoire diversity. Black lines represent the best fit line with the equation, with the *r*^2^ values described adjacently. Medians and interquartile ranges are depicted.

Next, we tested whether the decrease in naive T cells was related to the reduction in thymic output by assessing the thickness, anteroposterior, craniocaudal, and transverse lengths of thymus on high-resolution computed tomography (CT) scans (Fig. 3A). Thymoma was not found in any of the patients. Notably, smaller and hypodense thymi were observed among Freiburg 1a and Freiburg 1b patients when compared with the age-matched published reference range (median thymic thickness: Freiburg 1a = 0.38 cm, Freiburg 1b = 0.30 cm). However, the Freiburg group 2 patients were noted to have normal thymic thickness (median: 0.73 cm) (Fig. 3B) (21). Our data showed that all thymic dimensions positively correlated with repertoire diversity (Fig. 3C). Hence the loss in thymic volume is supportive of a decrease in thymic output, leading to a peripheral reduction in naive T cells and repertoire diversity in a proportion of CVID patients.

### Reduction in repertoire diversity exacerbates infection outcome in CVID

**FIGURE 4.**
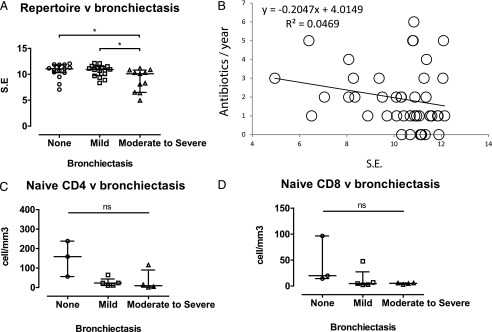
Repertoire diversity and infection burden. The effect of repertoire diversity on infection burden was assessed by correlating to the severity of bronchiectasis, use of antibiotics, and peripheral blood naive CD4 and CD8 counts. (**A**) Bronchiectasis was subclassified into none (open circle white), mild (open square), or moderate-to-severe (open triangle) according to the CT scan findings (*n* = 42). Repertoire diversities by S.E. of various bronchiectatic groups are shown. (**B**) The number of courses of antibiotics required by patients is plotted against the S.E. Black lines represent the best fit line with *r*^2^ values described adjacently. (**C**) Peripheral blood naive CD4 and CD8 T cell counts in various bronchiectatic groups are presented. Medians and interquartile ranges are depicted. Statistical differences were determined by two-tailed Mann–Whitney *U* test. *p < 0.05.

In view of the reduced repertoire diversity and naive T cells, we further hypothesized that there would be limited cognate T cells for T-dependent Ab responses, resulting in more frequent infections. To test this, we examined repertoire diversity against the severity of bronchiectasis and annual requirement for antibiotics, with the former graded as none, mild, or moderate-to-severe according to the CT findings. Reduction in repertoire diversity was observed in those patients with moderate-to-severe bronchiectasis (median S.E.: mild bronchiectasis = 11.0 [*p* = 0.953], moderate-to-severe bronchiectasis = 10.1 [*p* = 0.012]) (Fig. 4A). Similarly, patients with reduced repertoire diversity required more courses of antibiotics per year (Fig. 4B). However, we observed no statistical differences in peripheral blood naive CD4^+^ and CD8^+^ counts among the various bronchiectatic groups (Fig. 4C, 4D), indicating that the qualitative assessment of T cells by repertoire diversity may be a more superior predictor for infection risk than mere numerical T cell counts in CVID patients.

### Unique TCR amino CDR3 sequences in CVID patients

**FIGURE 5.**
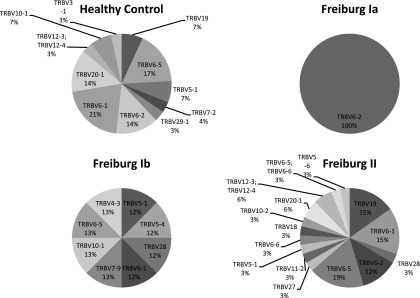
V-gene usage of subgroup unique TCR sequences in healthy donors and CVIDs. Thirty-three unique shared amino acid CDR3 sequences to healthy donors, 26 to the Freiburg group 2 patients, 6 to the Freiburg group 1b patients, and 3 to the Freiburg group 1a patients (*n* = 7) were identified. The variation in V-gene usages of the unique shared sequences of subgroups is shown in the pie charts. Freiburg 1a patients expressed 100% usage of TRBV6-2 in their unique shared sequence pools.

Finally, given the autoimmune association of CVID, we explored whether shared unique TCR amino acid CDR3 sequences could be found among patients. A TCR amino acid CDR3 sequence that occupied >0.01% of the repertoire was considered a significant immune response in an individual. All significant sequences were then cross-searched between all participants. Our search identified 445 sequences shared by more than one individual within our cohort of healthy donors and patients. Of these 445 sequences, 33 were unique to the healthy donors (*n* = 15), 26 to the Freiburg group 2 patients (*n* = 16), and 6 to the Freiburg group 1b patients (*n* = 8), but only 3 were unique to the Freiburg group 1a patients (*n* = 7). Interestingly, all 3 Freiburg 1a unique sequences (100%) commenced with the TRBV 6-2 gene segment despite no enrichment of this V-gene in the repertoire when weighted and unweighted V-gene usage analyses were performed. In contrast, the healthy donors, Freiburg 1b, and Freiburg group 2 patients exhibited a greater diversity in the range of V-gene segment expressed in their unique sequence pools (Fig. 5). However, the significance of these observations is not clear.

## Discussion

This study demonstrated that a restriction in T cell repertoire diversity is predominantly found in Freiburg group 1a patients, but not in other CVID patients. In addition, our data support that this reduction in repertoire diversity is in part due to a decrease of naive T cells and may worsen infection rate and bronchiectasis. Despite it being recognized that thymic output gradually declines with age, it is known to be active well into the seventh decade, and our data suggest the progressive loss in repertoire diversity in Freiburg 1a patients far exceeds the expected physiological rate (22). However, the loss of repertoire diversity by subtle clonal memory T cell expansion, as opposed to a decrease of naive T cells, cannot be completely ruled out. Although the larger TCRβ sequence pools and T cell memory compartments on immunophenotyping were normal, the higher cumulative frequency of the top 10 CDR3 sequences in Freiburg group 1a patients would support this notion.

Nevertheless, the collective decrease in TCR repertoire diversity, naive T cells, and thymic volume was consistent with orthogonal evidence supporting thymic failure in CVID patients (3, 23). Molecular studies have revealed that the numbers of copies of TCR excision circles were significantly reduced and CD31^+^ recent thymic immigrants were lower in CVID patients when compared with healthy donors (10, 24, 25). Moreover, other thymic-derived populations including naive, regulatory, and invariant NK T cells were all reported in lower frequencies in patients with CVID (14, 23, 26, 27). Although there is heterogeneity in the classification systems used in these studies, it is not surprising to note that the majority of defects were described in patients with low class-switched memory B cells, a high proportion of CD21^lo^ B cells, splenomegaly, and autoimmune cytopenias, thus gravitating this phenomenon toward the Freiburg 1a classification (15).

Although the T cell counts were low in all the CVID patients, different pathological mechanisms may be involved between subgroups. A number of intrinsic and extrinsic factors such as stress and infections are known to influence the thymic function. In mice, although infection with *Salmonella* resulted in transient thymic atrophy because of the loss in thymocytes, recovery was seen postinfection (28, 29). Similar observations have been made in rabies, HIV, *Mycobacterium*, tularemia, and *Listeria* infections, although these are uncommon in CVID (30, 31). Our data are currently unable to differentiate primary thymic failure from secondary causes. Given that low naive T cell counts were seen in all CVID patients, the difference in their TCR repertoire diversities may actually stem from the memory compartment. The cumulative frequencies of the top 10 CDR3 sequences, as well as weighted JSD V-gene analysis, actually hinted a masked oligoclonality memory compartment, which may only be apparent in patients with sufficient loss of naive T cells. However, our analyses were carried out on whole PBMCs, which is a major weakness of this study. To address this more accurately, future experiments should aim at analyzing purified naive and memory T cells to pinpoint the source of oligoclonality.

The progressive loss in repertoire diversity and thymic function are in line with the late onset of the disease. The lack of cognate T cells by primary or secondary primary thymic dysfunction may impair T-dependent Ab responses, thus exacerbating infection control. Conforming to this, the failure of humoral immunity also occurs later in life for the majority of patients. In the future, it would be of interest to examine whether arrest in thymic output coincides with the timing of humoral failure.

Although unweighted V-gene usage analysis suggested that thymic TCR synthesis was relatively normal in all patients, weighted V-gene usage analysis suggested that V-gene selection for clonal T cell expansion may be specifically altered in Freiburg 1a patients. This finding is consistent with previous studies showing preferential V-gene usage in CVID patients (3, 4, 6, 32). Moreover, our data revealed a highly restrictive set of TRBV 6-2 unique CDR3 sequences in Freiburg 1a patients. Although interesting, the relevance of these sequences is currently speculative. Without the knowledge of the patients’ HLA types, it is not feasible to determine their putative Ag targets. Furthermore, a number of these sequences may be Ag independent. Future work involving high-throughput TCR sequencing on enriched T cell subpopulations or cloned T cells with the knowledge of the full αβ conformation and patients’ HLA types is required to better characterize the biophysical characteristics of these unique CDR3 sequences. The emergence of standardized, multiparametric bioinformatics tools, such as VDJtools (33), is likely to further our understanding in this field. Finally, there is a need to strengthen the observation made between infection rate and repertoire diversity with more robust data because the current conclusion may be subjected to reporting bias from incomplete hospital records.

In summary, primary thymic dysfunction appears to contribute significantly to the TCR repertoire defect in Freiburg group 1a CVID patients. The progressive nature of CVID is highlighted in this study. Clinically, our findings emphasize the integration of combined B and T cell testing to identify those patients with the greatest risk for infection, particularly when the revised European Society for Immunodeficiencies (2014) diagnostic criteria now demand a more comprehensive assessment of the T cell compartment during the diagnostic workup. Future studies should aim at purifying and examining the memory T cell compartment in greater detail, as well as attempting to determine the putative T cell targets in CVID.

## Supplementary Material

Data Supplement
