# Supplementary material for: Accelerated Loss of TCR Repertoire Diversity in Common Variable Immunodeficiency
Source: J Immunol. 2016 Aug 1;197(5):1642–9. doi: 10.4049/jimmunol.1600526 (PMC4991247; doi:10.4049/jimmunol.1600526)
Supplement: Data Supplement [file JI_1600526.zip › JI_1600526_Supplemental_Material_1.pdf]

Patient no.	Age	Gender	Age of diagnosis	IgG	IgA	IgM	†Disease complications	Group	‡%smB	¶%CD21 lo
40	32	F	28	0.55	<0.05	0.2	Bronchiectasis, lymphadenopathy, ILD	B<1%	NA	NA
20	40	M	36	<0.1	0.07	<0.1	Bronchiectasis, splenomegaly, lymphadenopathy, AIC, ILD, enteropathy	B<1%	NA	NA
29	45	F	34	2.85	<0.05	<0.10	Bronchiectasis, splenomegaly, deranged LFTs, ILD	B<1%	NA	NA
30	60	M	21	Not known	Not known	Not known	Bronchiectasis, splenomegaly, lymphadenopathy, uveitis, AIC, enteropathy, renal cell carcinoma	B<1%	NA	NA
31	60	M	42	4.35	0.27	0.29	Mild deranged LFTs	B<1%	NA	NA
25	60	M	46	Not known	Not known	Not known	Bronchiectasis, splenomegaly, lymphadenopathy, nodular regenerating hyperplasia (liver), AIC, enteropathy	B<1%	NA	NA
21	65	F	59	0.87	<0.1	<0.1	Bronchiectasis, sarcoiliitis, deranged LFTs	B<1%	NA	NA
39	28	M	28	2.83	0.1	0.29	Splenomegaly, lymphadenopathy, AIC, ILD	Ia	0.08	42.2
13	33	M	19	5.29	<0.05	<0.03	None	Ia	0.36	31.3
14	45	M	Not known	Not known	Not known	Not known	Mild lymphadenopathy	Ia	0.01	62.3
23	45	F	42	2.91	<0.10	0.18	Splenomegaly, AIC	Ia	0.00	33.8
32	50	M	Not known	Not known	Not known	Not known	Splenomegaly, AIC, ILD	Ia	0.02	89.3
19	60	F	48	2.11	<0.07	0.05	Bronchiectasis, deranged LFTs, hypothyroidism, enteropathy	Ia	0.00	23.9
28	63	M	53	2.71	0.06	0.46	Bronchiectasis, Sjorgren's, AIC, enteropathy	Ia	0.01	28.7
15	17	F	8	1.84	<0.05	<0.06	Borderline splenomegaly	Ib	0.14	8.1
41	20	F	20	2.39	0.19	0.09	Splenomegaly, lymphadenopathy, iritis, ILD, enteropathy	Ib	0.05	10.6
16	24	F	23	0.11	<0.05	<0.10	Bronchiectasis	Ib	0.04	5.5
34	40	F	33	0.13	<0.05	0.1	Bronchiectasis, splenomegaly, lymphadenopathy, deranged LFTs, AIC, ILD	Ib	0.00	18.7
3	41	M	18	Not known	Not known	Not known	Bronchiectasis	Ib	0.09	0.5
37	50	F	36	3.81	0.14	0.35	Bronchiectasis, splenomegaly, lymphadenopathy, deranged LFTs, AIC, ILD	Ib	0.01	9.3
10	50	M	48	0.43	0.39	0.13	Bronchiectasis	Ib	0.30	5.1
5	60	F	43	2.94	0.08	0.31	B12 deficiency	Ib	0.12	5.1
12	65	M	55	4.27	1.64	0.26	Bronchiectasis	Ib	0.03	1.1
1	20	F	18	4.36	0.16	0.19	Mild alopecia areata	II	0.69	2.3
4	22	M	20	3.91	0.42	1.44	Bronchiectasis	II	1.03	0.5
18	23	M	14	0.35	<0.10	0.1	Bronchiectasis	II	3.11	9.8
6	27	F	23	3.09	<0.05	<0.10	None	II	0.43	34.8
11	28	F	24	1.65	0.17	0.18	None	II	0.76	2.6
17	33	M	29	<0.10	<0.05	<0.10	None	II	0.74	12.9
8	38	F	30	3.1	4.77	1.23	Bronchiectasis	II	0.66	21
27	40	F	38	0.4	<0.10	<0.10	Bronchiectasis, splenomegaly, AIC	II	1.30	66.9
33	41	F	38	1.87	<0.05	0.19	Bronchiectasis, transverse myelitis, ILD	II	0.49	6.3
26	45	F	40	1.75	<0.1	<0.1	Bronchiectasis, autoimmune diabetes, enteropathy	II	2.37	6.8
22	45	M	44	1.59	<0.05	0.11	Bronchiectasis, autoimmune diabetes, gastric cancer	II	1.68	9.9
36	45	M	44	3.46	0.48	0.44	Lymphadenopathy, ILD	II	0.76	9.8
9	50	M	49	3.92	0.06	0.16	Bronchiectasis	II	1.67	11.2
24	57	M	50	Not known	Not known	Not known	Bronchiectasis, splenomegaly, lymphadenopathy, optic neuritis	II	0.48	63.6
2	62	M	38	Not known	Not known	Not known	Bronchiectasis	II	0.56	0.5
7	67	F	60	2.55	<0.30	<0.10	Breast cancer	II	0.51	15.1
38	45	F	Not known	Not known	Not known	Not known	Bronchiectasis, splenomegaly, Nodular regenerative hyperplasia (liver), AIC, ILD, enteropathy	NK	NK	NK
35	50	F	42	Not known	Not known	Not known	Splenomegaly, lymphadenopathy, polyarteritis, deranged LFTs, AIC, ILD	NK	NK	NK
42	65	M	Not known	Not known	Not known	Not known	Bronchiectasis, ILD	NK	NK	NK

Supplemental Table I. Patient characteristics.

†Liver function test (LFT), autoimmune cytopenia (AIC) & interstitial lung disease (ILD) are abbreviated.
‡%sm = frequency of class-switched memory B-cells of total peripheral blood lymphocytes.
¶%CD21^{lo} = frequency of CD21^{lo} B-cells of total B-cells.

‡Participant (HC/ Patient)	Total sequencing read	†DNA clonotype (sequence read)	†In-frame productive AA clonotype (sequence read)	†Hyper-expanded clonotype (sequence read)	†Large clonotype (sequence read)	†Medium clonotype (sequence read)	†Low frequency clonotype (sequence read)
HC1	5944046	245831 (5759349)	194048 (2358996)	3 (265055)	463 (450030)	7237 (323050)	186345 (962502)
HC2	2589681	243113 (2325699)	186740 (1980973)	1 (24032)	424 (322404)	13489 (571200)	172826 (1441359)
HC3	2875091	329199 (2666132)	255089 (2291293)	3 (98331)	579 (421711)	7957 (382211)	246550 (1078720)
HC4	2654090	244574 (2463618)	194043 (2153237)	2 (88531)	214 (141372)	16878 (581454)	176949 (1284839)
HC5	2544491	195008 (2329550)	147740 (2000637)	7 (443129)	405 (295864)	10303 (478357)	137025 (1028363)
HC6	2881515	314398 (2637565)	243121 (2258466)	0 (0)	520 (242605)	12149 (575351)	230452 (1354948)
HC7	2570996	209900 (2262862)	159620 (1938837)	4 (182273)	374 (309077)	13973 (542758)	145269 (1111681)
HC8	2798398	273680 (2615470)	199012 (2096196)	6 (564393)	266 (196833)	6826 (309463)	191914 (1160764)
HC9	2643169	146774 (2485302)	121083 (2245713)	7 (347083)	395 (630431)	11182 (502838)	109499 (863159)
HC10	2804459	344261 (2609742)	269945 (2231453)	1 (33307)	446 (361042)	6249 (305241)	263249 (1453647)
HC11	2639502	242213 (2317974)	189054 (2021297)	1 (30503)	272 (276385)	14668 (562811)	174113 (1421593)
HC12	2730014	339116 (2506443)	260668 (2145789)	1 (27506)	202 (97890)	9779 (324692)	250686 (1488749)
HC13	2928688	242660 (2697497)	190516 (2343512)	1 (67686)	480 (459344)	10685 (494333)	179350 (1237103)
HC14	2767410	164546 (2591775)	129146 (2172904)	4 (451966)	456 (420347)	11611 (550441)	117075 (894682)
HC15	2850215	266187 (2630600)	211650 (2317436)	0 (0)	367 (222469)	13812 (554649)	197471 (1244179)
P40	2824351	380469 (2631829)	300586 (2297449)	0 (0)	315 (152721)	11042 (514565)	289229 (1630163)
P20	2412194	111181 (2235377)	85819 (1953483)	5 (141686)	611 (336697)	23681 (939424)	61522 (535676)
P29	2582440	73552 (2407924)	57242 (1949539)	9 (958946)	244 (223016)	11320 (411873)	45669 (355704)
P30	3294470	444027 (3016540)	344162 (2517314)	1 (91948)	241 (307877)	4655 (240470)	339265 (1877019)
P31	2950116	243578 (2707041)	188609 (2344563)	0 (0)	651 (316099)	14815 (818998)	173143 (1209466)
P25	2735874	127009 (2475015)	98718 (2103246)	4 (153284)	556 (401204)	20382 (857049)	77776 (691709)
P21	2899998	250472 (2707028)	198607 (2400529)	2 (225372)	273 (283657)	11154 (492184)	187178 (1399316)
P1	2668230	317897 (2462581)	250835 (2143195)	1 (22724)	122 (83269)	12442 (382213)	238270 (1654989)
P4	2543900	167594 (2305093)	127556 (1972544)	2 (47535)	331 (166290)	26391 (933847)	100832 (824872)
P18	2891905	264591 (2679068)	210453 (2323961)	0 (0)	180 (129194)	16501 (616653)	193772 (1578114)
P6	2867417	296827 (2595718)	223150 (2179692)	2 (57949)	276 (193830)	12541 (451945)	210331 (1475968)
P11	2610787	242969 (2411491)	188623 (2061329)	3 (108345)	498 (369445)	11385 (472671)	176737 (1110868)
P17	2819563	180356 (2614332)	140948 (2165449)	8 (327469)	429 (750661)	5877 (262200)	134634 (825119)
P8	2717774	335768 (2525147)	262633 (2160848)	0 (0)	247 (212867)	9938 (390573)	252448 (1557408)
P27	2384819	207803 (2130898)	147007 (1698313)	0 (0)	412 (213227)	21609 (663319)	124986 (821767)
P33	2612730	270644 (2417568)	208397 (2064735)	4 (155960)	335 (272813)	10487 (399776)	197571 (1236186)
P26	2733438	132977 (2312898)	106950 (1916914)	5 (186580)	670 (521361)	14256 (560768)	92019 (648205)
P22	2883111	182498 (2640817)	141063 (2362359)	5 (275535)	301 (436889)	15428 (610777)	125329 (1039158)
P36	2847694	267518 (2610515)	211439 (2300501)	4 (170011)	332 (427692)	8903 (412960)	202200 (1289838)
P9	2846165	301557 (2620897)	236305 (2249138)	2 (97520)	273 (198934)	10633 (460151)	225397 (1492533)
P24	2661790	135770 (2464787)	106276 (2143678)	1 (63678)	507 (320781)	23527 (1018056)	82241 (741163)
P2	2587562	133095 (2400220)	102737 (2055429)	5 (168315)	530 (613563)	15273 (575126)	86929 (698425)
P7	2932875	112121 (2622930)	84967 (2142554)	9 (629213)	466 (483833)	10449 (479658)	74043 (549850)
P39	2364596	99318 (2106684)	74539 (1805852)	7 (532618)	430 (354607)	12609 (459566)	61493 (459061)
P13	3034821	254507 (2761440)	196311 (2373782)	1 (34100)	325 (451905)	12454 (480823)	183531 (1406954)
P14	3150351	131942 (3010194)	102808 (2667591)	9 (1211284)	274 (462887)	5240 (266695)	97285 (726725)
P23	2528473	161677 (2312408)	123673 (1978167)	4 (95214)	567 (471403)	16545 (622590)	106557 (788960)
P32	2721391	194259 (2499390)	151486 (1949703)	3 (205550)	256 (351312)	13735 (425563)	137492 (967278)
P19	3497908	155834 (3229154)	121797 (2569695)	8 (410155)	800 (1102525)	6331 (410487)	114658 (646528)
P28	3278911	117219 (3175702)	89654 (2914674)	4 (1975078)	131 (151485)	3748 (241187)	85771 (546924)
P15	2475464	258756 (2271690)	196805 (1911064)	0 (0)	189 (71865)	18112 (565630)	178504 (1273569)
P41	2285897	118076 (2103553)	92636 (1821191)	1 (49773)	673 (363273)	22859 (853381)	69103 (554764)
P16	2499915	218594 (2308929)	179944 (2089209)	2 (110091)	374 (226825)	15204 (565698)	164364 (1186595)
P34	2228749	139904 (2031085)	108471 (1719221)	0 (0)	544 (282972)	24676 (805388)	83251 (630861)
P3	3354893	87551 (3199772)	68154 (2838709)	17 (1245195)	440 (1007415)	2382 (168892)	65315 (417207)
P37	2294874	144263 (2114421)	110579 (1782523)	0 (0)	176 (123785)	31344 (1020950)	79059 (637788)
P10	2882518	97442 (2384899)	73770 (2028208)	8 (537376)	499 (526617)	11584 (493082)	61679 (471133)
P5	2767362	167161 (2538338)	127457 (2149646)	2 (63013)	650 (342970)	20291 (852406)	106514 (891257)
P12	2541479	343181 (2350324)	271365 (2070009)	1 (22814)	86 (61582)	10809 (356628)	260469 (1628985)
P38	2130747	95924 (1962687)	72456 (1620332)	1 (16731)	806 (390189)	23481 (842964)	48168 (370448)
P35	2675665	234840 (2419974)	174796 (2062909)	1 (23893)	358 (261306)	17130 (671705)	157307 (1106005)
P42	2590663	192023 (2373187)	146386 (2014348)	2 (49664)	472 (322693)	17473 (679146)	128439 (962845)

Supplemental Table II. Details of sequencing output.

‡HC = healthy control, P = patient, hyper-expanded clonotype = >1% of repertoire, large clonotype = 0.01-1% of repertoire, medium clonotype = 0.001-0.01% of repertoire, low frequency (small) clonotype = <0.001% of repertoire.

†Clonotype count (sequence read)

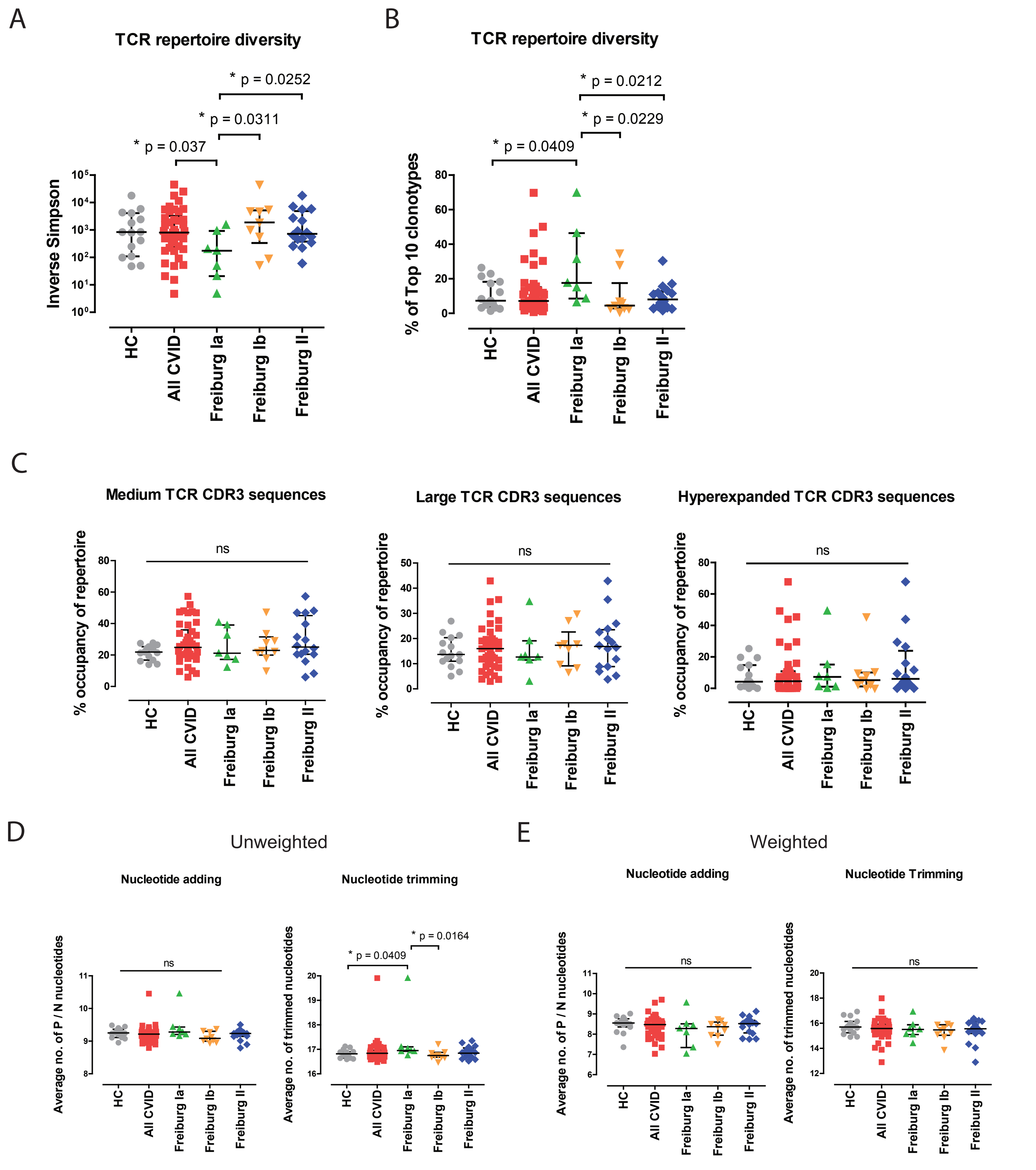

Supplemental Fig. 1. TCR repertoire diversity, VDJ P-segment and relative occupancies of medium, large and hyperexpanded amino acid TCRβ sequences in CVID. (A-B) Repertoire diversity by inverse Simpson's diversity index and frequency sum of the top 10 sequences of healthy donors (n=15, gray), all CVID patients (n=42, red), Freiburg group1a patients (n=7, green), group1b patients (n=9, orange) and group2 patients (n=16, blue) are presented. (C) The percentage occupancies of the repertoire of medium (0.001-0.01%), large (0.01-1%) and (>1%) amino acid TCRβ sequences are presented. (D-E) The average numbers of P and N nucleotide insertions (P3'V, N-region, N1-region, P5'D, P3'D, N2-region and P5'J) and V, D, J segment deletions (3' V-region trimmed, 5' D-region trimmed, 3' D-region trimmed and 5' J-region trimmed) of CDR3 sequences were determined according to the IMGT's HighV-Quest tool. Unweighted (left) and weighted (right) analyses are shown. The medians and interquartile ranges are depicted. Statistical differences are highlighted by *, ** or *** (two tailed Mann-Whitney U test).

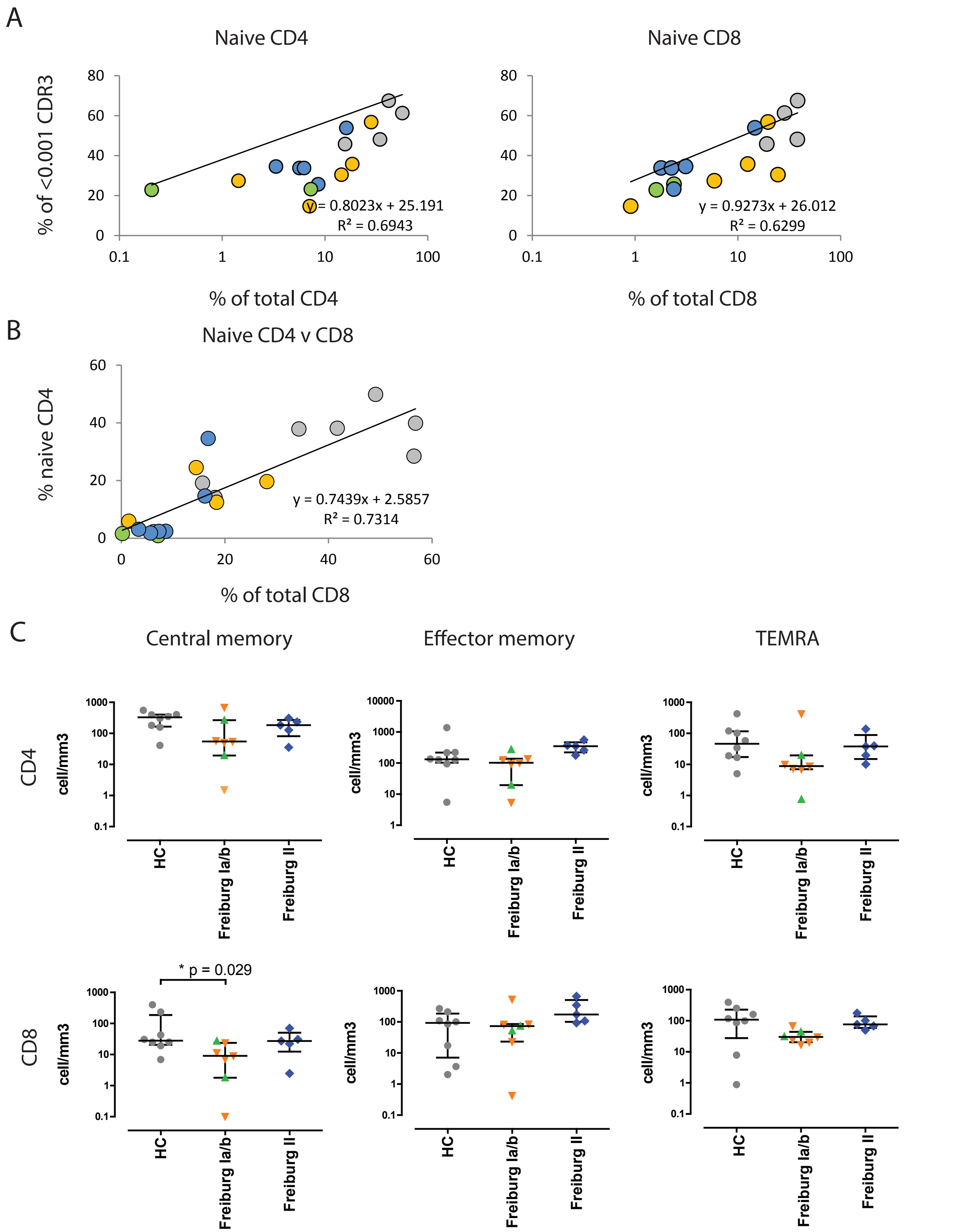

Supplemental Fig. 2. T-cell immunophenotyping and memory T-cell subpopulation counts in CVID. (A) The frequencies of naïve CD4 and CD8 T-cells are plotted against the percentage occupancy of low frequency (small) TCRβ CDR3 sequences. The black lines represent the best fit line with r^2 depicted adjacently. (B) The frequencies of naïve CD4 T-cells of total CD4 T-cells are plotted against the frequencies of naïve CD8 T-cells of total CD8 T-cells. (C) Peripheral blood T-cell subpopulations were enumerated utililzing counting beads by multi-parametric flow cytometry. The central memory (CM:$CCR7^+CD45RA^-CD28^+CD27^+$), effector memory (EM:$CCR7^-$ $CD45RA^-$) and TEMRA ($CCR7^-CD45RA^+$) CD4 and CD8 T-cells counts of the healthy donors (n=8, grey), Freiburg group1a patients (n=2, green), group1b patients (n=5, orange) and group2 patients (n=5, blue) are shown. The medians and interquartile ranges are depicted. Statistical differences are highlighted by *, ** or *** (two tailed Mann-Whitney U test).
